# Association between clinicopathologic characteristics and BRAF^V600E^ expression in Chinese patients with Langerhans cell histiocytosis

**DOI:** 10.1111/1759-7714.13179

**Published:** 2019-08-23

**Authors:** Hui Huang, Tao Lu, Yuxin Sun, Shan Li, Ji Li, Kai Xu, Rui e Feng, Zuo jun Xu

**Affiliations:** ^1^ Department of Pulmonary and Critical Care Medicine, Peking Union Medical College Hospital Chinese Academy of Medical Sciences & Peking Union Medical College Beijing China; ^2^ Pathological Department, Peking Union Medical College Hospital Chinese Academy of Medical Sciences & Peking Union Medical College Beijing China; ^3^ Radiological Department, Peking Union Medical College Hospital Chinese Academy of Medical Sciences & Peking Union Medical College Beijing China

**Keywords:** BRAF, Langerhans cell histiocytosis, mutation, pulmonary, V600E

## Abstract

**Background:**

The identification of V‐raf murine sarcoma viral oncogene homolog B1 (BRAF)^V600E^ mutations has been recommended in patients with Langerhans cell histiocytosis (LCH) with difficult diagnosis and failure of first‐line treatment. The reported frequencies of BRAF^V600E^ mutations vary in Chinese patients with LCH.

**Methods:**

We conducted a retrospective analysis of LCH patients with a definitive pathological diagnosis who were hospitalized between 2013 and 2017. The BRAF^V600E^ mutations were detected with the human BRAF^V600E^ amplification refractory mutation system‐PCR (ARMS‐PCR) kit from the collected tissue samples.

**Results:**

This study consisted of 46 male (68.7%) and 21 female (31.3%) patients, with a mean age of 29.1 years (range, 2–76 years). Most were adults (45/67.2%) with the multisysytem‐LCH (MS‐LCH) disease subtype (49/61.3%). The overall frequency of BRAF^V600E^ mutations was 22.4% (15 of 67 patients), confirmed by PCR analysis. These mutations were not closely correlated with age (nonadults vs. adults = 5/22.7% vs. 10/22.2*%*, *P* = 0.54), gender (female vs. male = 9/19.6% vs. 6/28.6*%*, *P* = 0.61), LCH classification type (single system: MS‐risk organ^+^: MS‐risk organ^−^ = 3/16.7%: 12:28.6%: 0, *P* = 0.19) or prognosis (cured: improved/stable: exacerbated: died = 4/44.4%: 19.2%: 20%: 0, *P* = 0.37). There were 33 patients (49.2%) with lung involvement, and 12 patients (36.3%) underwent lung biopsies; after screening, four patients were diagnosed with solitary pulmonary LCH, all of whom were negative for BRAF^V600E^ mutations.

**Conclusion:**

The BRAF^V600E^ mutation rate in patients with LCH was lower than those reported in other studies. In addition, BRAF^V600E^ mutations might not be correlated with age, gender, LCH classification type or prognosis for Chinese cases.

## Key points

### Significant findings of the study

The overall frequency of BRAF^V600E^ mutations in our study was lower than in some other reports. All four of our pulmonary Langerhans cell histiocytosis (PLCH) cases had negative BRAF^V600E^ mutation. BRAF^V600E^ mutations might not be correlated with age, gender, LCH classification type or prognosis for Chinese cases.

### What this study adds

The BRAF^V600E^ mutation rate in LCH varies.

## Introduction

As V‐rf murine sarcoma viral oncogene homolog B1 (BRAF)^V600E^ mutations are present in approximately half the samples from patients with Langerhans cell histiocytosis (LCH), and treatment with BRAF inhibitors has been reported to be effective for some patients with LCH, the identification of BRAF^V600E^ mutations is recommended for all patients with LCH with difficult diagnosis and failure of first‐line treatment (grade C2).^1^ The reported frequencies of BRAF^V600E^ mutations in patients with LCH vary among different ethnicities or countries.^2^ No BRAF^V600E^ mutations were reported in adults with LCH in the study by Tong *et al*.,^3^ but in the studies by Wei *et al*. and Zeng *et al*.,^4^, ^5^ the frequencies of BRAF^V600E^ mutations were 17.3% and 15.6% for Chinese adult patients with LCH and 46.4% and 32% for all recruited patients with LCH, respectively. Here, we retrospectively analyzed BRAF^V600E^ mutations and the clinical features of patients with LCH with a positive pathological diagnosis at our hospital over the last five years.

## Methods

### Patients

A computer‐assisted search for patients hospitalized at Peking Union Medical College Hospital from January 2013 to December 2017 identified 167 patients diagnosed with LCH according to the 2016 World Health Organization criteria.^1^ Most patients were diagnosed by our pathologist in consultation with biopsy/surgical samples from other hospitals. Finally, 67 patients with complete medical records, radiologic images and pathological specimens were retrospectively recruited into this study. All patients were followed‐up every one to six months, depending on disease activity and treatment. The mean follow‐up period was 36.8 months, ranging from seven to 59 months.

All patients underwent chest CT scans, cerebral magnetic resonance imaging, bone scintigraphy scans, whole body bone plain films, and bone marrow biopsies, and 26 patients underwent ^18F^fluorodeoxyglucose (FDG) positron emission tomography (PET) scans. The involved sites were defined according to the typical imaging scans and/or the pathological manifestations.

The following information was analyzed: age, sex, clinical manifestations, serological results, radiologic findings, pathological manifestations, treatments and outcomes. Hematoxylin‐eosin (HE) staining and immunohistochemical (IHC) staining analysis of CD68, CD1a, CD207, and S100 were performed for all enrolled patients. All glass slides were reviewed and scored by two pathologists (R.E.F. and J.L.), who were blinded to the molecular results. The two pathologists independently came to a consensus diagnosis based on the WHO recommendations for all enrolled patients.^6^

All patients and/or their relatives provided written informed consent. This study was approved by the ethics committee of Peking Union Medical College Hospital (JS‐1127, ZS‐1058), in accordance with the Declaration of Helsinki.

### DNA extraction

Tumor DNA was extracted from formalin‐fixed, paraffin‐embedded tissues from our pathological sample bank. Following HE and immunohistochemical staining, the samples with the highest CD1a‐positive histiocyte density were selected for further BRAF mutation analysis.

DNA was extracted from the collected tissue samples using the QIAGEN QIAamp DNA FFPE Tissue Kit (154051332, QIAGEN China (Shanghai) Co. Ltd., Shanghai, China), following the manufacturer's protocol.

### Detection of BRAF^V600E^ mutations by PCR analysis

The BRAF^V600E^ mutations were detected with the human BRAF^V600E^ ARMS‐PCR kit (P216010801Y, Amoy Diagnostics Co. Ltd., Xiamen, China), which has been approved by the China Food and Drug Administration. The extracted DNA quality was evaluated by amplification of a housekeeping gene following the instructions in the HEX channel. PCR was performed on the PCR System 7500 (ABI) system for 47 cycles according to the instructions supplied with the BRAF^V600E^ ARMS‐PCR kit. Both negative and positive controls were included in each set of amplifications. The sequencing results were analyzed and interpreted according to the manufacturer's protocol.

### Statistical analysis

Data were analyzed using the Statistical Analysis System (SAS) version 9.0 software package. Quantitative variables are presented as the mean ± standard deviation (SD), and categorical data are presented as the frequency and percentage in the text and figures.

## Results

### General clinical characteristics

The general clinical characteristics of the 67 enrolled patients are summarized in Table 1. The study group consisted of 46 male (68.7%) and 21 female (31.3%) patients, with a mean age of 29.1 years (range, 2–76 years). Most were adults, with 22 (32.8%) younger than 18 years and two (3.0%) older than 60 years. According to the 2016 WHO classification criteria for LCH, 18 patients (28.7%) had Single system‐LCH (SS‐LCH) type, seven (10.4%) had multisystem‐LCH (MS‐LCH) risk organ (RO)^+^ type and 42 (62.9%) had MS‐LCH‐RO^−^ type.

**Table 1:** General clinical characteristics for all enrolled cases

Case	Gender	Age (years)	Involved organ/tissue	Subtypes	Biopsy site	BRAF^V600E^ status	Treatment	Outcomes
1	M	43	Lung	SS	VATS lung biopsy	WT	Quit smoking	Cured
2	M	15	Pituitary	SS	surgical pituitary biopsy	WT	Radiotherapy	Improved
3	M	17	Maxillary sinus	SS	surgical maxillary sinus mass biopsy	WT	Surgery	Cured
4	M	22	Lung, pituitary, mandible, vertebrae	MS‐RO^−^	VATS lung biopsy, mandible biopsy	WT	Chemotherapy	Relapse
5	F	36	Pituitary, mandible	MS‐RO^−^	Surgical pituitary biopsy	WT	Radiotherapy	Improved
6	F	2	Skull, pituitary	MS‐RO^−^	Surgical skull biopsy	V600E	Chemotherapy	Improved
7	F	12	Lung, liver, pituitary	MS‐RO^+^	Surgical hepatic biopsy	WT	Chemotherapy	Improved
8	M	51	Lung, pituitary	MS‐RO^−^	VATS lung biopsy	WT	Chemotherapy	Improved
9	M	73	Lymph nodes, skin	MS‐RO^−^	Surgical lymph node biopsy	WT	Chemotherapy	Improved
10	M	12	Left humerus	SS	Surgical bone biopsy	WT	Surgery, radiotherapy	Improved
11	M	55	Lung, oral mucosa, lymph node	MS‐RO^−^	Surgical mucosa biopsy	V600E	Chemotherapy	Improved
12	M	37	Femur	SS	Surgical bone biopsy	WT	Surgery	Cured
13	M	26	Lung, vertebrae, liver, pituitary	MS‐RO^+^	Surgical bone and liver biopsy	WT	Chemotherapy, liver transplantation	Relapse
14	M	15	Lung, pituitary, thyroid	MS‐RO^−^	Surgical thyroid resection	WT	Surgery, chemotherapy	Improved
15	M	39	Pituitary	SS	Surgical pituitary biopsy	WT	Radiotherapy, surgery	Improved
16	M	13	Pituitary	SS	Surgical pituitary biopsy	WT	Surgery	Improved
17	M	37	Lung, thyroid	MS‐RO^−^	Thyroid fine needle biopsy	WT	Chemotherapy	Improved
18	M	5	Vertebrae	SS	Surgical bone biopsy	V600E	Surgery	Cured
19	F	6	Vertebrae	SS	Surgical bone biopsy	V600E	Surgery	Cured
20	F	9	Pituitary	SS	Surgical pituitary biopsy	WT	Surgery, radiotherapy	Improved
21	F	14	Pituitary, vertebrae, skin	MS‐RO^−^	Surgical bone biopsy	WT	Surgery, chemotherapy	Improved
22	F	25	Pituitary	SS	Surgical pituitary biopsy	WT	Surgery, radiotherapy	Improved
23	F	43	Skull	SS	Surgical bone biopsy	WT	Surgery, chemotherapy	Improved
24	M	76	Multiple lymph nodes	MS‐RO^−^	Surgical lymph node biopsy	WT	Chemotherapy	Exacerbation
25	M	42	Lung, mandible	MS‐RO^−^	Surgical mandible biopsy	V600E	Chemotherapy	Improved
26	M	17	Skull, femur, pituitary	MS‐RO^−^	Surgical bone biopsy	V600E	Chemotherapy	Improved
27	M	12	Skull, femur, vertebrae, pituitary	MS‐RO^−^	Surgical bone biopsy	WT	Chemotherapy	Improved
28	M	43	Pituitary, vertebrae, tibia	MS‐RO^−^	Surgical bone biopsy	V600E	Chemotherapy	Improved
29	M	12	Vertebrae	SS	Surgical bone biopsy	WT	Surgery, radiotherapy	Improved
30	F	49	Lung, pituitary, liver, lymph node	MS‐RO^+^	VATS lung biopsy, skin biopsy	WT	Chemotherapy	Improved
31	M	28	Lung, pituitary, skin	MS‐RO^−^	VATS lung biopsy	WT	Chemotherapy	Improved
32	F	17	Lung, pituitary, skin, lymph node	MS‐RO^−^	Skin biopsy	WT	Chemotherapy	Relapse
33	M	21	Lung, thyroid, pituitary, liver, lymph node	MS‐RO^+^	Thyroid fine needle biopsy	WT	Chemotherapy, thyroid radiotherapy	Improved
34	M	15	Pituitary, liver, lymph node	MS‐RO^+^	Liver fine needle biopsy	WT	Chemotherapy	Improved
35	M	8	Lung, thyroid, pituitary, lymph node	MS‐RO^−^	Thyroid fine needle biopsy	WT	Chemotherapy	Improved
36	M	24	Lung, skull, pituitary, skin, stomach	MS‐RO^−^	Stomach and skin biopsy	V600E	Chemotherapy	Exacerbation
37	F	14	Skull, pituitary	MS‐RO^−^	Surgical bone biopsy	WT	Surgery, chemotherapy	Improved
38	M	20	Pituitary, skin	MS‐RO^−^	Skin biopsy	WT	Surgery	Improved
39	M	31	Lung, skin, muscle, lymph node	MS‐RO^−^	Surgical muscle biopsy	WT	Chemotherapy	Improved
40	M	7	Skull, radius, vertebrae, pituitary	MS‐RO^−^	Surgical bone biopsy	WT	Surgery, chemotherapy	Improved
41	M	39	Skull, vertebrae, lung	MS‐RO^−^	Surgical bone biopsy	WT	Chemotherapy	Improved
42	F	32	Skin, lymph node, liver	MS‐RO^+^	Lymph node biopsy	WT	Chemotherapy	Improved
43	M	14	Lung, pituitary	MS‐RO^−^	Surgical pituitary biopsy	WT	Chemotherapy	Improved
44	F	23	Skull	MS‐RO^−^	Surgical bone biopsy	V600E	Surgery	Cured
45	F	52	Rib	MS‐RO^−^	Surgical bone biopsy	V600E	Surgery	Cured
46	M	38	Lung, skin, pituitary, thyroid	MS‐RO^−^	Thyroid fine needle biopsy	WT	Chemotherapy	Relapse
47	M	28	Lung, alveolar bone	MS‐RO^−^	VATS lung biopsy, bone biopsy	WT	—	Died
48	F	12	Lung, pituitary	MS‐RO^−^	Surgical pituitary biopsy	WT	Chemotherapy	Improved
49	M	42	Lung	SS	VATS lung biopsy	WT	Quit smoking	Cured
50	F	32	Lung, pituitary	MS‐RO^−^	VATS lung biopsy	WT	—	Stable
51	F	25	Skull, vertebrae, skin	MS‐RO^−^	Skin biopsy	WT	Chemotherapy	Improved
52	M	16	Lung, pituitary	MS‐RO^−^	Transbronchial lung biopsy	V600E	Chemotherapy	Improved
53	M	23	Lung, skull, pituitary, thyroid	MS‐RO^−^	Thyroid fine needle biopsy	WT	Chemotherapy	Improved
54	M	54	Vertebrae, pituitary	MS‐RO^−^	Surgical bone biopsy	WT	Chemotherapy	Improved
55	F	47	Lung, femur, pituitary, thyroid	MS‐RO^−^	Surgical thyroid resection	V600E	Surgery, chemotherapy	Improved
56	M	31	Lung, skull, pituitary, skin	MS‐RO^−^	Skin biopsy	V600E	Chemotherapy	Improved
57	F	31	Femur, skull, vertebrae, rib, oral mucosa, pituitary	MS‐RO^−^	Oral mucosa biopsy	V600E	Chemotherapy	Improved
58	M	25	Pituitary	SS	Surgical pituitary biopsy	WT	Surgery, radiotherapy	Improved
59	F	24	Lung, thyroid, pituitary	MS‐RO^−^	Thyroid fine needle biopsy	WT	Chemotherapy	Improved
60	M	57	Lung, liver, lymph nodes	MS‐RO^+^	Surgical lymph node biopsy	WT	Chemotherapy	Improved
61	M	24	Skull, vertebrae, pituitary	MS‐RO^−^	Surgical bone biopsy	WT	Chemotherapy	Improved
62	M	27	Lung, pituitary	MS‐RO^−^	VATS lung biopsy	WT	Chemotherapy	Improved
63	M	47	Lung	SS	VATS lung biopsy	WT	Quit smoking	Cured
64	M	47	Skull	SS	Surgical bone biopsy	V600E	Chemotherapy	Improved
65	M	29	Skull, pituitary	MS‐RO^−^	Surgical bone biopsy	WT	Chemotherapy	Improved
66	M	35	Lung	SS	VATS lung biopsy	WT	Quit smoking	Improved
67	F	48	Lung, pituitary	MS‐RO^−^	Surgical pituitary biopsy	WT	Chemotherapy	Improved

F, female; M, male; MS, multisystem; RO, risk organ; SS, single system; VATS, video‐assisted thoracic surgery; WT, wild type.

The BRAF^V600E^ molecular analysis was successful for all 67 enrolled patients. The overall frequency of BRAF^V600E^ mutations was 22.4% (15 of 67 patients) according to the PCR analysis. The main involved sites included the pituitary gland (42/62.7%), lung (33/49.3%), skull (16/23.9%), vertebrae (14/20.9%), lymph nodes (11/16.4%), skin (11/16.4%), thyroid gland (8/11.9%), limb bone (8/11.9%), and liver (7/10.4%). Most patients (50/74.6%) improved after surgery, chemotherapy and/or radiotherapy. Nine patients (13.4%) were cured after surgical resection (6/9%) or after quitting smoking (3/4.5%). Four patients (4/6%) relapsed, and two patients (2/3%) experienced exacerbation of the disease, although they underwent chemotherapy. One patient (1/1.5%) was stable without treatment, and one (1/1.5%) died of respiratory failure.

### Clinical characteristics of patients with different BRAF^V600E^ statuses

The clinical characteristics of patients with different BRAF^V600E^ mutation statuses are shown in Table 2. These mutations were not closely correlated with age (nonadults vs. adults = 5/22.7% vs. 10/22.2%, *P* = 1.00), gender (female vs. male = 9/19.6% vs. 6/28.6%, *P* = 0.61), LCH classification type (SS: MS‐RO^+^: MS‐RO^−^ = 3/16.7%: 12:28.6%: 0, *P* = 0.19), or prognosis (cured: improved/stable: exacerbated: died = 4/44.4%: 19.2%: 20%: 0, *P* = 0.37). As the pituitary gland, lung and thyroid were the most common associated organs in our cohort, the associations between the involvement of these organs and BRAF^V600E^ mutation status were analyzed. However, there were no differences in the prevalence of the BRAF^V600E^ mutation between patients with involvement of the pituitary gland, lung and thyroid and patients with other involved sites. In addition, all the LCH patients with liver involvement were negative for BRAF^V600E^ mutations. There were no correlations between BRAF^V600E^ mutations status and the prognosis.

**Table 2:** The clinical characteristics of patients with different BRAF^V600E^ mutations

	Characteristics		Mutation type (*n*)	Wild type (n)	*P*‐value
Children	Gender	Male	3	11	1.00
		Female	2	6	
	LCH subtype	SS	2	6	1.00
		MS‐RO^+^ & MS‐RO^−^	3	9	
	Lung involvement	Yes	1	6	1.00
		No	4	11	
	Pituitary involvement	Yes	3	15	0.21
		No	2	2	
	Thyroid involvement	Yes	0	2	1.00
		No	5	15	
	Prognosis	Cured/improved/stable	5	16	1.00
		Exacerbation/relapse/died	0	1	
Adults	Gender	Male	6	26	0.63
		Female	4	9	
	LCH subtype	SS	1	9	0.53
		MS‐RO^+^ & MS‐RO^−^	9	26	
	Lung involvement	Yes	5	21	0.84
		No	5	14	
	Pituitary involvement	Yes	5	19	1.00
		No	5	16	
	Thyroid involvement	Yes	1	5	1.00
		No	9	30	
	Prognosis	Cured/improved/stable	9	30	1.00
		Exacerbation/relapse/died	1	5	

LCH, Langerhans cell histiocytosis; MS, multisystem; RO, risk organ; SS, single system.

### Patients with LCH with pulmonary involvement

Pulmonary involvement is frequently present in systemic forms of LCH. In addition, pulmonary LCH (PLCH) is restricted to the lungs.^7^ Among the 67 patients with LCH in this study, there were 33 patients (49.2%) who had lung involvement. In addition, 12 patients (36.3%) underwent lung biopsies. After multiple screening tests, only four patients (12.1%) were diagnosed with PLCH, and none were positive for BRAF^V600E^ mutations. The clinical characteristics of patients with or without lung involvement LCH are shown in Table 3. Lung involvement was more common in patients with the MS‐LCH subtype (*P* = 0.006), and patients with thyroid gland involvement (100% vs. 36.2*%*, *P* = 0.0073). After either surgical or fine needle biopsy, eight patients were diagnosed with LCH with thyroid involvement. The imaging studies of these eight patients showed classic lung shadows indicating diffuse cysts with bizarre shapes.

**Table 3:** The clinical characteristics of patients with or without lung involvement LCH

	Characteristics		Lung involvement (*n*)	Without lung involvement (*n*)	*P*‐value
Children	Gender	Male	4	10	1.000
		Female	3	5	
	LCH subtype	SS	0	8	0.022
		MS‐RO^+^ & MS‐RO^−^	7	7	
	BRAF^V600E^status	WT	6	11	1.000
		MT	1	4	
	Pituitary involvement	Yes	7	11	0.263
		No	0	4	
	Thyroid involvement	Yes	2	0	0.091
		No	5	15	
	Prognosis	Cured/improved/stable	6	15	0.318
		Exacerbation/relapse/died	1	0	
Adults	Gender	Male	21	11	0.094
		Female	5	8	
	LCH subtype	SS	4	6	0.354
		MS‐RO^+^ & MS‐RO^−^	22	13	
	BRAF^V600E^status	WT	21	14	0.840
		MT	5	5	
	Pituitary involvement	Yes	14	10	0.936
		No	12	9	
	Thyroid involvement	Yes	6	0	0.071
		No	20	19	
	Prognosis	Cured/improved/stable	21	18	0.359
		Exacerbation/relapse/died	5	1	

LCH, Langerhans cell histiocytosis; MS, multisystem; RO, risk organ; SS, single system; WT, wild type.

In our cohort, most of the patients with LCH with lung involvement were patients with systemic LCH. All four patients with PLCH were smokers. Three were cured after quitting smoking and avoiding secondhand smoke for four to six months, without medications. The fourth patient with PLCH was diagnosed three months prior to this manuscript being written, and his lung infiltrations improved after quitting smoking, without taking medication. Although all patients with PLCH had good prognoses, there was no difference in prognosis between patients with LCH with and without lung involvement.

## Discussion

The BRAF gene is located on chromosome 7q34, and is a member of the RAF kinase family. Although mutations of BRAF have been identified in a large number of solid tumors, it was first reported by Badalian‐Very *et al*. in 2010 that BRAF^V600E^ expression was identified in 57% of samples from patients with LCH.^8^ Following this study, there were several studies that focused on the mitogen‐activated protein kinase (MAPK) pathway signal transmission, including BRAF^V600E^,^2^, ^8^, ^9^, ^10^, ^11^, ^12^, ^13^ mitogen‐activated protein kinase 1 (MAP2K1) and NRAS.^14^, ^15^, ^16^, ^17^ It was reported that the activating BRAF^V600E^ mutation rate ranged from 35% to 60% in different studies using PCR or immunohistochemistry (IHC) staining^2^, ^8^, ^9^, ^10^, ^11^, ^12^ in patients with LCH. According to the review by Selway *et al*. the BRAF^V600E^ mutation rate was 51.13% in 397 patients with LCH.^18^ The mutation rate varies across different studies and different races. The BRAF^V600E^ mutation rates reported in Chinese patients with LCH were 0 in the study by Tong *et al*. ^3^ 56% in the study by Wei *et al*. and 22.5% in our study.^4^

The patient age distribution, involved sites, stage, and different detection tests might influence the BRAF^V600E^ subtype.^3^, ^8^, ^19^ Badalian‐Very *et al*.^8^ and Héritier *et al*.^13^ reported that younger LCH patients tended to be positive for BRAF^V600E^ mutations, and most patients with BRAF^V600E^ mutations in the study by Wei *et al*. had bone involvement or the MS‐LCH subtype^4^; in the study by Tong *et al*. ^3,4,8^ skin and lung were the most commonly involved sites, and 77.8% of the patients had the LCH‐SS subtype. However, according to the meta‐analysis of existing LCH BRAF^V600E^ studies by Selway *et al*. there was no difference in the presence of BRAF^V600E^ mutations between adults and children, between those with the SS‐LCH and MS‐LCH subtypes, and those with different involved sites.^18^ In our cohort, BRAF^V600E^ mutations were not correlated with age, gender, LCH classification type, or prognosis. According to the meta‐analysis by Selway *et al*. and the study by Heritier *et al*. on the BRAF^V600E^ mutation in patients with LCH,^13^, ^18^ the rate of the BRAF^V600E^ mutation was increased in patients who experienced involvement of higher risk organs, such the liver and spleen. In the study by Selway *et al*.,^18^ although 75% of the biopsied liver samples were positive for BRAF^V600E^ mutations, none of our patients with LCH with liver involvement had BRAF^V600E^ mutations.

The lung is a commonly involved site in patients with LCH, and PLCH has been commonly reported in previous studies. However, only four patients (6%) in our cohort were diagnosed with solitary PLCH. Most of the previous studies did not show the completed screening tests for the enrolled patients with LCH.^2^, ^4^, ^8^, ^10^, ^11^, ^12^, ^13^, ^14^, ^15^, ^16^ In our study, all 67 patients underwent strict screening tests including chest CTs, cranial MRIs, bone scans and bone marrow biopsies, with the exception of detailed serum tests. In addition, 26 patients underwent ^18F^FDG‐PET‐CT scans, which is a useful and sensitive tool for the identification of active lesions, the stratification of disease stages, and the monitoring of a therapeutic response in patients with LCH.^20^ Most of our patients with lung involvement were diagnosed with the MS‐LCH subtype after these screening tests, and only four patients were diagnosed with solitary PLCH.

Solitary PLCH is a smoking‐related non‐neoplastic disease, and the study by Yousem *et al*. failed to show clonality in patients with PLCH.^21^ However, some studies reported BRAF^V600E^ mutation rates ranging from 28% to 89% in patients with PLCH.^22^ In our study, all four PLCH patients smoked, and all were negative for BRAF^V600E^ mutations. Three of them were cured, and the fourth patient experienced improvement after quitting smoking and avoiding secondhand smoke, without medications. The natural history of PLCH varied widely. Some patients with PLCH may remit or stabilize after quitting smoking. However, others may develop pulmonary fibrosis, pulmonary hypertension, and respiratory failure, even after chemotherapy. There has been no further analysis of the BRAF^V600E^ mutation status for those self‐cured PLCH patients in most studies.

There were several limitations in our study. First, all enrolled patients had a definitive diagnosis of LCH and had complete clinical records, radiological images and pathological specimen, which could cause a selection bias. The BRAF^V600E^ mutations in our study were detected by PCR analysis, and the DNA was extracted from formalin‐fixed paraffin‐embedded tissues, which could minimize the sensitivity. Second, not all enrolled patients underwent lung biopsies. There were 33 patients (49.2%) who had lung involvement, but only 12 patients (36.3%) underwent lung biopsies. LCH was diagnosed with extrapulmonary tissue biopsies, even for some cases with diffuse pulmonary infiltrations. However, for these cases, the clinical characters and extrapulmonary pathological manifestations were sufficient for the diagnosis of LCH, and to avoid excessive damage, lung biopsies were not performed. Third, peripheral blood BRAF^V600E^ mutations for LCH cases had been previously reported, and the mutation status might be associated with the disease burden and therapy response.^23^, ^24^, ^25^, ^26^ As our study was retrospective, we were unable to obtain samples from all enrolled cases, and most of them were treated when we connect with them. Peripheral blood BRAF^V600E^ mutation status will be analyzed in our future prospective studies, especially for MS‐LCH cases.

In conclusion, the BRAF^V600E^ mutation rate in patients with LCH was lower than in some reported studies. In addition, BRAF^V600E^ mutations might not correlated with age, gender, LCH classification type, or prognosis in our patients with LCH.

## Disclosure

The authors do not have any competing interests and/or bias with regard to this publication.
